# Incipient resistance to an effective pesticide results from genetic adaptation and the canalization of gene expression

**DOI:** 10.1111/eva.13166

**Published:** 2020-12-14

**Authors:** Xiaoshen Yin, Alexander S. Martinez, Abigail Perkins, Morgan M. Sparks, Avril M. Harder, Janna R. Willoughby, Maria S. Sepúlveda, Mark R. Christie

**Affiliations:** ^1^ Department of Biological Sciences Purdue University West Lafayette IN USA; ^2^ Department of Anatomy, Cell Biology & Physiology Indiana University School of Medicine Indianapolis IN USA; ^3^ School of Forestry and Wildlife Sciences Auburn University Auburn AL USA; ^4^ Department of Forestry and Natural Resources Purdue University West Lafayette IN USA

**Keywords:** ATP synthase, contemporary evolution, genetic adaptation, pesticides, resistance, RNA‐seq

## Abstract

The resistance of pest species to chemical controls has vast ecological, economic, and societal costs. In most cases, resistance is only detected after spreading throughout an entire population. Detecting resistance in its incipient stages, by comparison, provides time to implement preventative strategies. Incipient resistance can be detected by coupling standard toxicology assays with large‐scale gene expression experiments. We apply this approach to a system where an invasive parasite, sea lamprey (*Petromyzon marinus*), has been treated with the highly effective pesticide 3‐trifluoromethyl‐4‐nitrophenol (TFM) for 60 years. Toxicological experiments revealed that lamprey from treated populations did not have higher survival to TFM exposure than lamprey from untreated populations, demonstrating that full‐fledged resistance has not yet evolved. In contrast, we find hundreds of genes differentially expressed in response to TFM in the population with the longest history of exposure, many of which relate to TFM’s primary mode of action, the uncoupling of oxidative phosphorylation, and subsequent depletion of ATP. Three genes critical to oxidative phosphorylation, *ATP5PB, PLCB1,* and *NDUFA9*, were nearly fixed for alternative alleles in comparisons of SNPs between treated and untreated populations (*F_ST_* > 5 *SD* from the mean). *ATP5PB* encodes subunit b of ATP synthase and an additional subunit, *ATP5F1B*, was canalized for high expression in treated populations, but remained plastic in response to TFM treatment in individuals from the untreated population. These combined genomic and transcriptomic results demonstrate that an adaptive, genetic response to TFM is likely driving incipient resistance in a damaging pest species.

## 1 INTRODUCTION

The evolution of resistance often occurs in response to the continued and large‐scale application of chemical compounds such as antibiotics, herbicides, and pesticides (Davies & Davies, 2010; Délye et al., 2013; Georghiou & Taylor, 1977; Gould et al., 2018). The costs associated with the evolution of resistance measure in the billions of dollars per year and cut across diverse fields ranging from health care to agriculture (Levy & Marshall, 2004; Pimentel & Burgess, 2014; Smith & Coast, 2013). Increased rates of disease, decreased food security, and large‐scale environmental costs represent only some of the negative effects when microbes, pathogens, and pests can no longer be effectively controlled (Gould et al., 2018; Ranson & Lissenden, 2016). In light of these costs, there is a considerable need to prevent or delay the evolution of resistance. Unfortunately, resistance is often detected too late, after most individuals in a population have acquired a resistant phenotype (McKenna, 2013). Once this stage is reached, there is little to be done except to implement alternative control measures, which are often less effective (Ghosh et al., 2019; Peterson et al., 2018), or stop employing control measures until resistant genotypes are eliminated from the population, which can take tens to hundreds of generations (Andersson & Hughes, 2010; Andersson & Levin, 1999; Christie et al., 2019).

One solution to mitigating the costs of resistance is early detection; identifying the evolution of resistance in its incipient stages can allow time for developing alternative control measures or implementing management actions that can prevent the onset of population‐wide resistance (Andow & Ives, 2002; Apple & Smith, 1976; Stenberg, 2017). One approach to detecting incipient resistance, which we define here as an early genetic or phenotypic response to treatment, is to screen a large fraction of the population for resistant genotypes or phenotypes. However, such measures can be costly and impractical because of the large sample sizes required to identify small numbers of fully resistant individuals. Detectability issues (e.g., false positives and negatives) compound this problem. Alternatively, the coupling of experimental, eco‐toxicological assays with the identification of genomic and transcriptomic responses in populations with varied treatment histories can provide key insights into how and whether resistance is beginning to evolve. We applied this design to an invasive vertebrate pest species that has been controlled with a chemical pesticide for 60 years.

**Figure 1:**
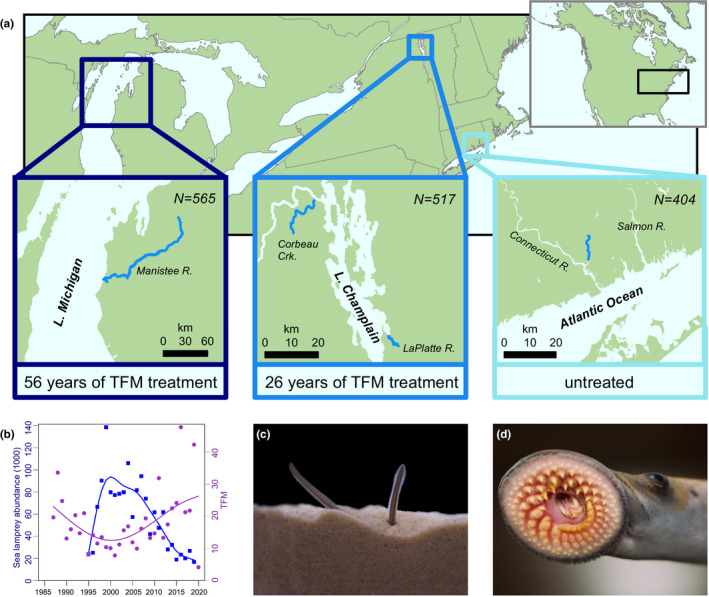
Sample collection sites, sample sizes, and pesticide (TFM) treatment histories for larval sea lamprey used in this study (a). Lake Champlain and Connecticut River sea lamprey were collected from their native range, whereas sea lamprey from Lake Michigan are considered invasive. Sea lamprey abundance (blue) and metric tons of TFM (purple) applied to Lake Michigan from 1987 onwards (b); notice that TFM is an effective pesticide with decrease in lamprey abundance following increases in TFM application. The larval stage of sea lamprey remains buried in the sediment (c), aggregates in high densities, and is targeted with the pesticide TFM. After transformation, the juveniles are generalist parasites, using their circularly arrayed teeth to attach to hosts and their rasping tongues to feed (d)

Throughout the Laurentian Great Lakes, invasive sea lamprey (*Petromyzon marinus*) have negatively impacted native fish communities by feeding parasitically on other fishes (Lawrie, 1970). With the construction and improvement of shipping canals in the early 1900s, sea lamprey expanded their range from the northwestern Atlantic Ocean to the Great Lakes (Smith & Tibbles, 1980) (Figure 1). Adult sea lamprey are hematophagous parasites of other fishes and are not host‐specific (Figure 1d). The lack of host specialization, coupled with ample adult‐spawning and larval‐rearing habitat throughout the Great Lakes region, spurred the growth of parasitic sea lamprey populations into the millions of individuals (Smith & Tibbles, 1980). High abundance of invasive sea lamprey in the Great Lakes contributed to rapid and substantial declines in native lake trout (*Salvelinus namaycush*) and other ecologically and commercially important fishes (Baldwin et al., 2002; Bronte et al., 2003). In response to the effects of sea lamprey on native fish and fisheries, there was an immediate and concerted effort to develop efficient means for controlling sea lamprey populations in the Great Lakes.

One effort, initiated in the 1950s, involved testing over 4,000 chemical compounds on sea lamprey and other fish species (Applegate, 1957). The organic compound 3‐trifluoromethyl‐4‐nitrophenol (hereafter, TFM) was found to effectively kill larval lamprey with few detectable effects on other fishes at low concentrations (Hubert, 2003; Middaugh et al., 2014; but see Marsden & Siefkes, 2019). The larval stage of sea lamprey (Figure 1c) is targeted because they are small (typically < 120 mm), aggregate at high densities, and encompass multiple year classes. The primary mode of action for TFM is to uncouple oxidative phosphorylation (Birceanu et al., 2009, 2011), and because sea lamprey are highly sensitive to this mode of action, TFM is a very effective pesticide (Figure 1b). Since the application of TFM to locations with abundant larval lamprey, invasive sea lamprey populations have declined by up to 90% (Heinrich et al., 2003; Smith & Tibbles, 1980). However, TFM applications kill most, but not all, larval sea lamprey (Dunlop et al., 2017) and it is possible that resistant individuals could survive and reproduce (Christie et al., 2019). If this process is repeated over enough generations, then resistant individuals could increase in frequency—a scenario documented in many systems where pests have been controlled by chemical means (Whalon et al., 2008). The evolution of resistance would greatly reduce the effectiveness of TFM, which is currently the primary control method for invasive sea lamprey (Dunlop et al., 2017), and decreased pesticide effectiveness would likely hamper the restoration of native and commercially important fish populations throughout the Great Lakes.

To test for the evolution of resistance, we first collected larval sea lamprey from three populations with varied histories of TFM treatment (Figure 1a): Lake Michigan (56 years of TFM treatment), Lake Champlain (26 years of TFM treatment), and Connecticut River (0 years of TFM treatment). Sea lamprey are possibly native to Lake Champlain (Bryan et al., 2005, Waldman et al., 2015, 2004), so all three populations not only have different durations of exposure to TFM but also have different evolutionary histories. Larvae were acclimated in a common environment for 4 months before being experimentally exposed to TFM at lethal, sublethal, and control (no TFM) levels. Detailed survival and toxicological analyses were coupled with tissue‐specific (i.e., muscle, liver, brain) RNA sequencing (RNA‐seq). Survival analyses revealed that the outright resistance of sea lamprey to lethal concentrations of TFM could not be detected, but genomic and transcriptomic analyses suggest that the early, incipient stages of resistance may be well under way in populations with a history of pesticide exposure.

## 2 MATERIALS AND METHODS

### 2.1 Sample collection and experimental design

We collected a total of 2,035 larval sea lamprey (ammocoetes) from five locations during summers of 2016 and 2017 using pulsed‐DC backpack electrofishers, focusing our sampling efforts on populations with different durations of exposure to TFM (Figure 1; Appendix S1: Table S1). In 2016, we obtained 565 larvae from the Manistee River in Lake Michigan, 517 ammocoetes from Corbeau Creek and the LaPlatte River in Lake Champlain, and 404 ammocoetes from Connecticut River (Figure 1). Lake Michigan ammocoetes have been treated with TFM since 1960 (Lavis et al., 2003), Lake Champlain ammocoetes have been treated with TFM since 1990 (Marsden et al., 2003), and Connecticut River has never been treated with TFM. Importantly, sea lamprey have very little natal philopatry (i.e., adults rarely spawn in their natal stream) and high population connectivity (Bjerselius et al., 2000; Bryan et al., 2005), meaning that TFM treatments applied in one part of the lake affect the development of resistance throughout the entire lake (Christie et al., 2019). To examine lamprey of similar ages and developmental stages, we only collected ammocoetes from a narrow size range (80–120 mm) and we only collected larvae that had never been previously exposed to TFM. All ammocoetes were collected within five calendar days (July 11–July 15, 2016) of each other and were transported to Purdue's Aquaculture Research Laboratory immediately after collection.

Upon arrival at the Aquaculture Research Laboratory at Purdue University, ammocoetes were acclimated in 24, 30‐gallon flow‐through holding tanks corresponding to population of origin at densities of 60–67 individuals per tank. Each tank received flow‐through well water and was lined with 10 cm of sand to allow ammocoetes to burrow. During acclimation, ammocoetes were fed a mixture of baker's yeast and water (1 gram of yeast per ammocoete) two times per week. Larval mortality in the holding tanks was minimal (<2.1% across all populations, *n* = 30 individuals) during the acclimation period. Phenotype (including gene expression) is a product of genetics and environment. Thus, larvae were intentionally maintained in a common environment for a long time (four months) to minimize the effect of different collection environments (Pespeni et al., 2013). Ideally, we would perform laboratory crosses to eliminate environmental effects (sensu Christie et al., 2016), but this study design was not practical given that it takes larvae an average of 3–6 years to reach sizes equivalent to individuals that are treated with TFM in the field. In 2017, we collected an additional 284 ammocoetes from the Platte River in Lake Michigan and 265 ammocoetes from Turner Falls, Massachusetts, to independently validate our 2016 toxicological analyses (Appendix S1: Tables S1 and S2). In comparison with 2016, we did not use these individuals for gene expression analyses and larvae were acclimated for only six weeks in order to better draw comparisons to wild larvae.

To examine lethal and sublethal effects, we constructed an experimental array to conduct exposure trials in a temperature‐controlled environmental chamber (12.8°C) consisting of 36, 2.5‐gallon glass aquaria. Each tank received freshwater (average temperature = 13.9°C, pH = 8.3) that was gravity‐fed from storage tanks on top of the array. Alkalinity within experimental tanks was maintained within a constant range across exposures (96–140 ppm) by mixing well water (alkalinity > 200 ppm) with water treated via reverse osmosis (alkalinity ~ 20 ppm). Each treatment tank also received an inflow line from one of two adjacent multi‐channel peristaltic pumps (Gilson MINIPULS Evolution; Fisherbrand FH100M). A single air stone was suspended in the center of each tank to provide aeration and promote mixing.

### 2.2 Lethal exposure trials

**Figure 2:**
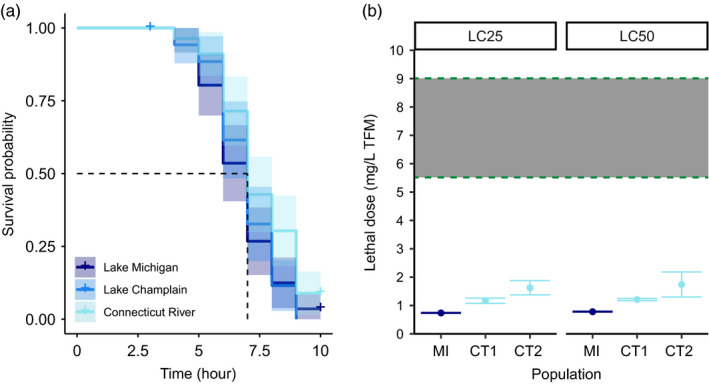
Results of toxicology assays illustrating survival through time and lethal concentrations. In 2016 trials, survival analyses revealed that previously unexposed larvae collected from treated populations did not survive at higher rates than larvae collected from the untreated population during exposure to lethal concentrations of TFM (a). In 2017 trials, independent toxicology assays from additional larvae collected from Lake Michigan (MI) and Connecticut River (CT) again revealed that larvae collected from populations with a history of exposure did not survive lethal doses of TFM at higher rates than larvae collected from the untreated population (b). Lethal concentrations for 25% (LC25) and 50% (LC50) mortality were calculated, and two separate assays were performed for Connecticut River individuals (CT1 and CT2). Dashed green lines border the range of TFM values larvae would be exposed to under typical stream application conditions. Collectively, these results illustrate that resistance to lethal concentrations of TFM has not yet evolved

In 2016 (2016 trials; Appendix S1: Table S2), we conducted two toxicological exposure trials to directly assess whether sea lamprey populations exhibited differential mortality in response to TFM exposure. We began each exposure by transferring ammocoetes from their holding tanks into randomized experimental array tanks prefilled with 7 L of water. For the first 2016 trial (TOX 1; Appendix S1: Table S2), each population was allotted 12 aquaria with seven ammocoetes per tank (*n* = 84 lamprey per population). Within a population, eight treatment aquaria received TFM (*n* = 56 individuals per population), while four control aquaria (*n* = 28 individuals per population) received a volume of water (control) equal to the volume of TFM distributed into treatment tanks. In TOX 1, we aimed for high (i.e., >80%) mortality to allow us to assess whether survivorship varied among populations and we challenged ammocoetes in treatment tanks with a TFM concentration of 3 mg/L, as determined by a multi‐concentration, small‐scale pilot exposure trial. We allowed ammocoetes to acclimate for a period of one hour to the experimental tanks before introducing TFM with peristaltic pumps. To simulate conditions associated with a typical TFM stream application, ammocoetes were exposed to TFM for ten hours followed by a two‐hour “washout period.” TFM concentrations were monitored hourly and verified by measuring TFM absorption using a spectrophotometer with readings taken at 400 nm based on standard curves created via serial dilutions the day of the experiment. Mortality was assessed hourly over the 12‐hr duration of the experiment. At the conclusion of the experiment, dead individuals were measured for weight and length. The second 2016 trial (TOX 2; Appendix S1: Table S2) had the same experimental setup as TOX 1, except that a TFM concentration of 1.67 mg/L was administered to treatment tanks to target more modest levels of mortality (Appendix S1: Table S2). When TFM is applied in the field, maximum stream concentrations of TFM range from 5.5 to 9 mg/L for the pH and alkalinity values used in our experimental treatments (Figure 2b).

To analyze survivorship data, we conducted a Kaplan–Meier survival analysis (Kaplan & Meier, 1958) that generates an estimate of survival probability (*S*) at time t_i_ and is calculated as follows:

(1) $$S\left(t_{i}\right)=S\left(t_{i}‐1\right)\left(1‐\frac{d_{i}}{n_{i}}\right)$$

where $S\left(t_{i}‐1\right)$ is the probability of being alive at time $t_{i}‐1$, *n_i_* is the number of individuals alive just prior to *t_i_*, and *d_i_* is the number of deaths occurring at *t_i_* (Kaplan & Meier, 1958). We performed the survival analysis in R (R core team, 2019), using the packages SURVIVAL and OIsurv to create survival curves and assess statistical differences between the three populations (Therneau, 2015, 2004); *p*‐values associated with pairwise tests used to determine which populations differ in their survivorship were FDR‐adjusted (Benjamini & Hochberg, 1995).

To independently confirm survival analyses from 2016 trials, we conducted three additional toxicological exposure trials with ammocoetes collected in 2017 (2017 trials; Appendix S1: Table S2). We conducted toxicological exposure trials similarly to the 2016 trials with three modifications to increase power for the detection of lethal concentrations in a given population. First, each trial examined only one population at a time. Second, trials lasted for 20 hr instead of 12 hr. Third, these trials consisted of five treatments—each with three replicate tanks of seven individuals—including the control (0, 0.5, 1.2, 1.5, and 2 mg/L of TFM), instead of a single TFM treatment (Appendix S1: Table S2). With this experimental design, we could quantify lethal concentrations (e.g., LC50). To compare differences in survival, we computed lethal concentrations for each trial using the DRC package (v 3.0–1) (Ritz et al., 2015) and again made comparisons among trials at different dosage levels using the Kaplan–Meier survival analyses (Kassambara & Kosinski, 2018; Therneau, 2015, 2004).

### 2.3 Sublethal exposure trials

To assess whether resistance was evolving at a sublethal level, we conducted two additional exposure trials (i.e., gene expression trials GE 1 and GE 2; Appendix S1: Table S2) to assess (a) how gene expression profiles changed in response to TFM and (b) whether TFM exposure altered patterns of gene expression differently among three populations with varying histories of exposure. The experimental design for sublethal exposure was similar to that for the toxicological exposure trials outlined above with two key differences. First, we exposed ammocoetes to sublethal levels of TFM (i.e., 0.3 mg/L in GE 1 and 0.2 mg/L in GE 2; Appendix S1: Table S2). Second, we preserved tissue from ammocoetes at a predetermined time point (*t* = hour 6) during the 12‐hr gene expression exposure trial for RNA‐seq. All populations were run concurrently, and all individuals were sampled on the same day at the same time such that any differences in gene expression could not be due to differences in circadian rhythms (Ruiz‐Jones & Palumbi, 2015). Sampling consisted of removing a single ammocoete per replicate tank and immediately euthanizing it in a lethal dose of MS‐222 before weighing, measuring, and flash‐freezing in cryovials with liquid nitrogen. At the completion of the experiment, cryovials were directly transferred to a −80°C freezer.

### 2.4 RNA‐seq and differential gene expression analysis

We used tissue‐specific RNA‐seq to compare patterns of gene expression in ammocoetes from the three populations with varying histories of exposure to TFM. Comparing tissue‐specific patterns of expression required that we first dissect specific tissue samples from the body segments collected during gene expression trials. Given TFM’s established mode of action, we targeted ammocoete muscle, liver, and brain tissues for downstream analyses of gene expression profiles. We first transferred individuals frozen at −80°C into a solution of RNAlater‐ICE (Ambion Inc., Austin, TX, USA) at 10 volumes of solution relative to sample mass and allowed the solution to permeate into the tissue for 16 hr at −20°C. Once the solution had permeated into the tissue and segments had thawed, we dissected muscle, liver, and brain tissues for subsequent mRNA extractions (Appendix S1: Table S3). In total, we performed 70 mRNA extractions across three populations (Lake Michigan, Lake Champlain, and Connecticut River) and three tissue types (muscle, liver, and brain) using the Qiagen RNeasy Mini Kit (Appendix S1: Table S3). Library preparation and mRNA sequencing were performed by the Purdue Genomics Core Facility with samples from GE 1 and GE 2 (Appendix S1: Table S2) being sequenced on either an Illumina 2500 or an S2 NovaSeq lane. We obtained a total of 3.25 billion 150‐bp paired‐end reads, which we first used to create an annotated transcriptome (see “RNA‐seq, transcriptome assembly, and differential gene expression analysis” in *SI Materials and Methods* for details on transcriptome assembly).

We aligned all reads to our assembled sea lamprey transcriptome and estimated transcript abundances using RSEM (Li & Dewey, 2011), and identified differentially expressed genes (hereafter, DEGs) using a GLM with TFM concentration and sequencing machine as fixed effects in edgeR (McCarthy et al., 2012; Robinson et al., 2010). We compared gene expression profiles of treated versus untreated ammocoetes within each population to identify genes that were significantly up‐ and downregulated in response to TFM treatment. For genes to be classified as differentially expressed, we set minimum thresholds for significance (FDR correction via the Benjamini–Hochberg method; *p*‐value < .05). While we did not explicitly filter DEGs by log‐fold change, the minimum log2‐fold changes we observed for DEGs were > 2 across all three populations (2.04, 3.50, and 2.74 for Lake Michigan, Lake Champlain, and Connecticut River populations, respectively). We conducted the differential gene expression analysis at the gene level (i.e., collapsing isoforms) because gene‐level estimates tend to be more accurate, spread reads over fewer features, and incur lower multiple testing penalties compared with transcript‐level (i.e., not collapsing isoforms) estimates (Soneson et al., 2016). Finally, GOseq was used to determine which gene ontology functional categories were overrepresented among differentially expressed genes in TFM‐treated individuals for each population‐tissue combination (Young et al., 2010). Only functionally enriched categories significant below an FDR‐adjusted *p*‐value threshold of .05 were retained. We constructed gene ontology (GO) hierarchy networks of the top 100 significant GO terms identified with DEGs (FDR‐corrected *p*‐value < .01) using the metacoder package (Foster et al., 2017).

### 2.5 Genomic analysis

Using the trimmed RNA‐seq reads, we called SNPs following the joint genotyping workflow (Brouard et al., 2019) provided by the Genome Analysis Toolkit (GATK 3.8) (McKenna et al., 2010) for muscle (*n* = 43; Lake Michigan: *n* = 16, Lake Champlain: *n* = 13, Connecticut River: *n* = 14) and liver (*n* = 19; Lake Michigan: *n* = 7, Lake Champlain: *n* = 6, Connecticut River: *n* = 6) tissue samples separately (see “Genomic analysis” in *SI Materials and Methods* for details on SNP calling). To validate SNPs called from the GATK joint genotyping workflow with RNA‐seq reads, we also called SNPs following the RNA‐seq variant calling pipeline by supplying one sample to HaplotypeCaller at a time. We used the SNPs that were identified in both the GATK joint genotyping workflow and the RNA‐seq pipeline in all subsequent analyses. For a subset of 19 individuals, we sequenced both muscle and liver tissues. By independently calling SNPs with muscle and liver tissue samples from the same individuals, we were able to validate our SNP calling pipeline (i.e., we used genotypes called from liver tissue as a validation for those called from muscle tissue). Genotypes were identical between liver and muscle tissues at 93% of loci. For each individual, genotypes with fewer than five reads were set as missing values. Muscle samples from two individuals 401 (Lake Champlain) and 402 (Lake Michigan) (Appendix S1: Table S3) had over 90% missing data and thus were excluded from further analyses. Finally, loci genotyped across at least 80% of all samples were retained as high‐quality SNPs, and this resulted in SNPs called for 41 and 19 samples from muscle and liver tissues, respectively (Appendix S1: Table S3). In each population, we further excluded loci out of Hardy–Weinberg equilibrium, which we identified using the package HWxtest (likelihood ratio *p*‐value < .05) (Engels, 2009). We calculated pairwise *F_ST_* for each locus among all three populations (i.e., Lake Michigan versus Lake Champlain, Lake Michigan versus Connecticut River, and Lake Champlain versus Connecticut River populations) using Weir and Cockerham's unbiased estimator (Weir & Cockerham, 1984). To detect outliers, we z‐transformed *F_ST_* in each comparison and defined loci with *F_ST_* greater than five standard deviations from the mean as outlier loci (Axelsson et al., 2013; Willoughby et al., 2018). We identified genes corresponding to outlier loci using the sea lamprey genome (Smith et al., 2018) and identified gene functions according to the annotation available at https://genomes.stowers.org/organism/Petromyzon/marinus. In order to confirm our *F_ST_* outliers, we calculated allele frequency difference (*AFD*) (Berner, 2019) and compared it with *F_ST_*, and we conducted kNN‐based genome scans to test whether genetic differentiation between populations at outlier genes was driven by selection (Pfeifer et al., 2020).

## 3 RESULTS

### 3.1 TFM survival

The larvae collected from treated tributaries were not previously exposed to TFM prior to the toxicological assays (i.e., any resistance, if detected, could not be due to inducible tolerance (Hua et al., 2013)). In 2016 trials (TOX 1–2; Appendix S1: Table S2), the mortality for treated individuals was highly similar across all populations over a 12‐hr exposure period. The combined mortality for treated individuals was 96.4%, 100%, and 91.1% for Lake Michigan, Lake Champlain, and Connecticut River populations, respectively (Figure 2a). There was no mortality within the control group for any population. In our 2017 trials (TOX 3–5; Appendix S1: Table S2), survivorship through time again did not vary substantially among populations (Appendix S1: Figure S1) and estimates of lethal concentrations required to kill 50% of the population (LC50) varied only slightly among populations and trials (Figure 2b). The Lake Michigan population had lower estimated lethal concentration values for 25% and 50% mortality (LC25: mean 0.74 ± *SE* 0.01 mg/L; LC50: mean 0.78 ± *SE* 0.01 mg/L) than for larvae collected from Connecticut River (LC25: mean 1.39 ± *SE* 0.17 mg/L; LC50: mean 1.48 ± *SE* 0.24 mg/L) (Figure 2b). The lethal concentration values for the Connecticut River population were larger than that of the Lake Michigan population at both LC25 and LC50 values. Furthermore, no individuals from Lake Michigan survived TFM concentrations greater than 2 mg/L (Appendix S1: Figure S1). In total, we performed five independent toxicological trials with six tested concentrations and never detected any differences in survival consistent with resistance (Appendix S1: Table S2). If outright resistance has evolved, we would expect to observe higher survivorship in sea lamprey collected from populations with a history of treatment (i.e., Lake Michigan or Lake Champlain) in comparison with sea lamprey collected from areas that have never been treated (i.e., Connecticut River). We found no evidence to support this prediction.

### 3.2 Variation in gene expression

**Figure 3:**
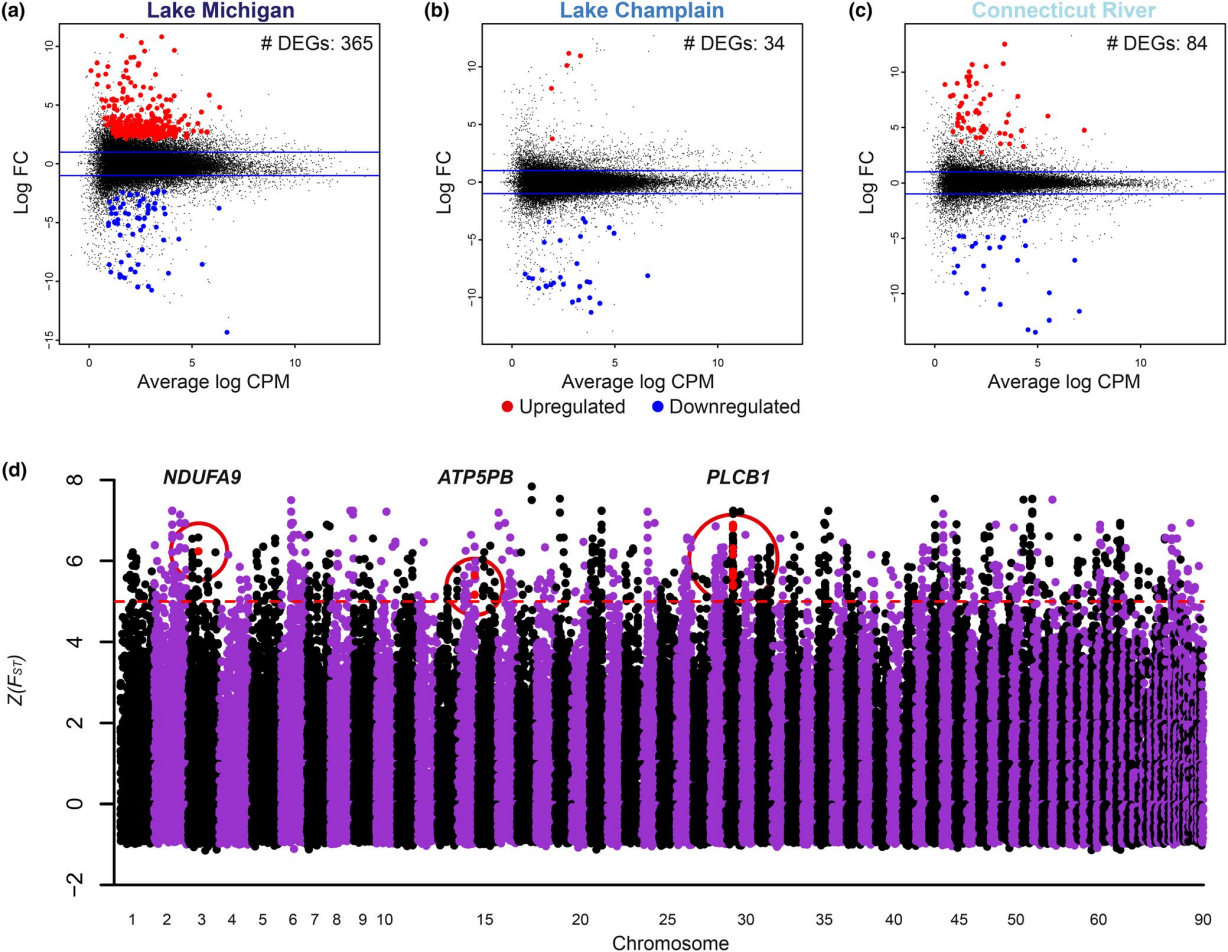
Transcriptomic and genomic evidence of incipient resistance (IR) in sea lamprey. A total of 365 genes were differentially expressed in response to sublethal concentrations of TFM in larval sea lamprey collected from Lake Michigan, the population with the longest history of TFM treatment (a). By contrast, only 34 and 84 genes were identified as differentially expressed in sea lamprey from Lake Champlain (b) and Connecticut River (c), respectively. After aligning reads back to the sea lamprey reference genome (sea lamprey have 99 chromosomes, 90 of which are assembled), calling SNPs, and calculating *F_ST_* between Lake Michigan and Connecticut River for muscle tissue samples, three outliers relating to TFM’s primary mode of action, uncoupling the electron transport chain, were identified: *NDUFA9*, a gene encoding a subunit of complex I in the electron transport chain, *ATP5PB*, a gene encoding subunit b of ATP synthase, and *PLCB1*, a gene encoding phospholipase c beta 1 (d)

**Figure 4:**
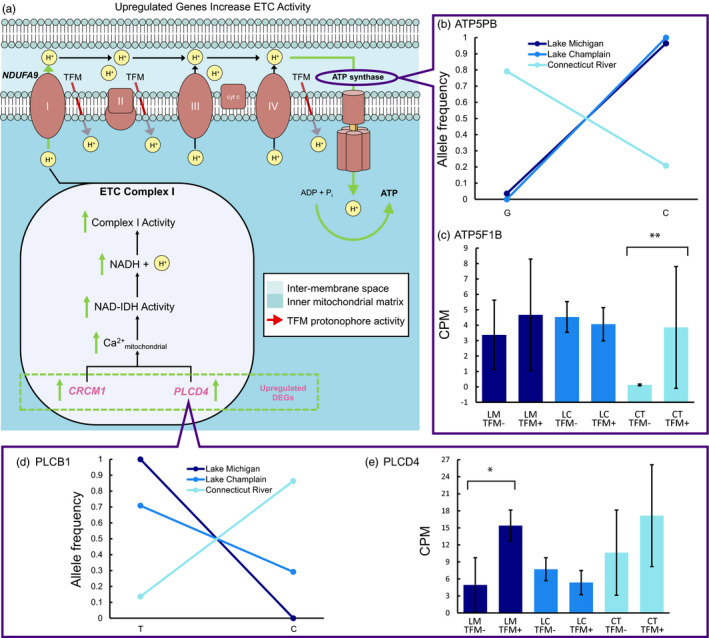
The pesticide TFM’s primary mode of action is to act as a protonophore, translocating protons from the inter‐membrane space back into the inner mitochondrial matrix, thus disrupting oxidative phosphorylation (a). *NDUFA9* (NADH:ubiquinone oxidoreductase subunit A9) encodes complex I in the electron transport chain (ETC) and was found in comparison between Lake Michigan and Connecticut River (a). Several genes upregulated in Lake Michigan, the population with the longest history of exposure, may play a role in responding to TFM’s mode of action. For example, the upregulation of *CRCM1* (calcium release‐activated calcium modulator 1) and *PLCD4* (phospholipase c delta 4) increases intracellular concentrations of calcium and likely enhance oxidative phosphorylation in Lake Michigan ammocoetes when exposed to TFM (a, e). The gene *ATP5PB* (ATP synthase peripheral stalk‐membrane subunit b), which encodes subunit b of ATP synthase and was identified as a putatively adaptive outlier (Figure 3d), is nearly fixed at a bi‐allelic SNP in the treated Lake Michigan and Lake Champlain populations, but segregates for different alleles in the untreated sea lamprey population (b). A gene encoding an additional subunit of ATP synthase, *ATP5F1B* (ATP synthase F1 subunit beta), is canalized for high expression in treated populations (i.e., the gene is expressed at high levels in both treatment (TFM+) and control (TFM‐) individuals from the treated Lake Michigan (LM) and Lake Champlain (LC) populations) (c). By contrast, this gene is not constitutively upregulated in untreated, Connecticut River (CT) sea lamprey, but responds plastically to treatment. The gene *PLCB1* (phospholipase c beta 1), which encodes phospholipase c beta 1 and was also identified as an outlier (Figure 3d), is nearly fixed for alternative alleles at a bi‐allelic SNP in the treated Lake Michigan and the untreated sea lamprey populations (d). A directly related gene encoding phospholipase c delta 4, *PLCD4*, shows significantly different levels of expression between control (TFM‐) and treatment (TFM+) in Lake Michigan (e). Error bars depict standard deviation, and *p*‐value was calculated from FDR‐corrected tests of differential gene expression

By contrast, we observed large differences in patterns of gene expression among sea lamprey from different populations exposed to the same, sublethal concentration of TFM. Remarkably, when comparing samples treated with 0.3 mg/L TFM to control samples, we found 365 genes that were differentially expressed (298 upregulated and 67 downregulated in comparison with control individuals) in muscle tissue in the Lake Michigan population, dwarfing the number of differentially expressed genes (DEGs) found in both Lake Champlain (*n* = 34) and Connecticut River (*n* = 84) populations (Figure 3a–c; Appendix S1: Table S4). No DEGs were shared among all three populations, only three DEGs were common to two of the three populations, and reduced population responses were observed when lower concentrations (i.e., 0.2 mg/L) of TFM were applied and when different tissues were examined (Appendix S1: Table S5, Figures S2–S4). Among the 365 DEGs detected in Lake Michigan, *CRCM1* (calcium release‐activated calcium modulator 1; see Appendix S1: Table S8 for full names) and *PLCD4* (phospholipase c delta 4) are directly related to TFM’s primary mode of action. By serving as a protonophore that transports protons across the inner mitochondrial membrane, TFM can uncouple oxidative phosphorylation and deplete ATP (Birceanu et al., 2009, 2011, 2014). *CRCM1* (LogFC = 2.60) encodes a calcium release‐activated channel protein that controls the influx of calcium into cells when depleted, and *PLCD4* (LogFC = 2.41) encodes an enzyme, phospholipase c delta 4, responsible for hydrolyzing phosphatidylinositol 4,5‐bisphosphate into two secondary messenger molecules, one of which (inositol 1,4,5‐trisphosphate or “IP3”) is known to release cellular calcium stores and increase calcium concentrations within the cell (Berridge, 1993). Calcium plays an integral role in activating and driving the oxidative phosphorylation cascade in mitochondria, and studies have demonstrated a positive relationship between nanomolar‐free calcium and the rate of phosphorylation (Fink et al., 2017; Glancy et al., 2013). Therefore, upregulated expression of genes that increase intracellular concentrations of calcium may enhance oxidative phosphorylation in Lake Michigan larvae and partially compensate for the uncoupling effects of TFM (Figure 4a). Additionally, in gene ontology (GO) hierarchy networks, we also found regulation of calcium ion transport, regulation of calcium ion transmembrane transport, regulation of calcium ion transmembrane transporter activity, and regulation of voltage‐gated calcium channel activity as significant biological processes (Appendix S1: Figure S5).

### 3.3 Genomic variation

Out of 513,815 SNPs detected with muscle tissue samples, 1,638 SNPs (corresponding to 119 outlier genes) were identified as outliers (Z(*F_ST_*)> 5) in the comparison between Lake Michigan and Connecticut River. Of those 119 outlier genes, three were directly related to TFM’s known mode of action (Birceanu et al., 2009, 2011, 2014). In comparison between Lake Michigan and Connecticut River, *ATP5PB* (ATP synthase peripheral stalk‐membrane subunit b) had an *F_ST_* value greater than five standard deviations from the mean (Figure 3d, Appendix S1: Figure S7). *ATP5PB* encodes a subunit of ATP synthase (subunit b) and, similar to many of the genes identified by expression analyses, appears to be responding to strong selection in order to restore normal oxidative phosphorylation in response to TFM’s primary mode of action (Figure 4a). Closer examination of the genotypes at the bi‐allelic SNP found in this gene illustrates that a single allele is nearly fixed in both Lake Michigan and Lake Champlain, but only occurs rarely in Connecticut River (Figure 4b). This outlier was also observed in comparisons between Lake Champlain and Connecticut River populations across tissue types (Z(*F_ST_*) = 4.73, 5.11, respectively; Appendix S1: Figure S8, Figure S10). Although the Connecticut River lamprey are not fixed at the genetic level for this variant, they were able to respond plastically with respect to TFM at ATP synthase. The gene *ATP5F1B* (ATP synthase F1 subunit beta), which codes for subunit beta of ATP synthase, was significantly upregulated in Connecticut River lamprey in response to TFM treatment (LogFC = 4.68, FDR‐corrected *p*‐value < .0072, Figure 4c) when compared to unexposed, control individuals, suggesting a plastic response. Interestingly, *ATP5F1B* was upregulated in both control and treatment individuals from Lake Michigan and Lake Champlain in comparison with Connecticut River control individuals, suggesting a genetic response to constitutively increase expression of this gene (Figure 4c).

An additional gene, *PLCB1* (phospholipase c beta 1), was identified as an outlier between Lake Michigan and Connecticut River populations and had an *F_ST_* value greater than six standard deviations from the mean (Figure 3d, Appendix S1: Figure S7). Similar to the differentially expressed *PLCD4* (LogFC = 2.41; Figure 4a), *PLCB1*, a nearly identical variant of *PLCD4*, encodes an enzyme catalyzing the production of inositol 1,4,5‐trisphosphate (“IP3”). Alternative alleles are almost fixed at this bi‐allelic SNP in Lake Michigan and Connecticut River populations, and similar patterns of allele frequency are observed in Lake Michigan and Lake Champlain (Figure 4d). This outlier was consistently detected in comparisons between Lake Michigan and Connecticut River across tissue types (Appendix S1: Figure S9). *PLCD4* was upregulated in treated individuals in Lake Michigan.

Lastly, *NDUFA9* (NADH:ubiquinone oxidoreductase subunit A9), an outlier gene encoding complex I in the electron transport chain (ETC) was found in comparisons between Lake Michigan and Connecticut River and had an *F_ST_* value greater than six standard deviations from the mean (Figure 3d, Appendix S1: Figure S7), but not in comparisons between Lake Champlain and Connecticut River. There was a high correlation between *F_ST_* and *AFD* across all three pairwise comparisons (Lake Michigan versus Lake Champlain: *r* ^2^ = 0.9423, Lake Michigan versus Connecticut River: *r* ^2^ = 0.9022, Lake Champlain versus Connecticut River: *r* ^2^ = 0.9418; Appendix S1: Figure S6) and the kNN‐based genome scans generated positive *deltaF_ST_* at outlier SNPs located on genes *NDUFA9*, *PLCB1*, and *ATP5PB*, suggesting that selection is driving the extreme genetic differentiation observed at these genes (Appendix S1: Figure S11). Furthermore, while the outlier SNPs located on *NDUFA9* and *ATP5PB* are located on introns, one outlier SNP located on *PLCB1* is a nonsynonymous substitution. The remaining 116 outlier genes (Figure 3d) had little to do with TFM’s known mode of action, but instead appear to be related to adaptation to the recently colonized environments.

## 4 DISCUSSION

Here, we provide evidence suggesting the evolution of incipient resistance in invasive sea lamprey treated with a pesticide. We found 365 genes differentially expressed (relative to controls from the same population) in response to TFM in larvae from Lake Michigan, the population with the longest history of using TFM (56 years). This transcriptional response represents a nearly fivefold greater number of differentially expressed genes in comparison with larvae collected from the Connecticut River population. Interestingly, several of the 365 DEGs detected (e.g., *CRCM1* and *PLCD4*) in Lake Michigan are directly related to TFM’s primary mode of action, uncoupling oxidative phosphorylation.

We also identified genomic evidence of a response to selection imposed by TFM. In comparisons between Lake Michigan and Connecticut River, large shifts in allele frequencies at *ATP5PB*, which codes for a subunit of ATP synthase, indicate a response to selection in a gene that is critical to successful oxidative phosphorylation (Figure 3d). A directly related gene, *ATP5F1B*, is constitutively expressed at high levels in treated populations but differentially expressed in the Connecticut River population, suggesting that an additional component of ATP synthase has responded to pesticide‐induced selection. Thus, the two populations with a lengthy history of TFM treatment show adaptive responses in ATP synthase at the genomic level, while the naïve, Connecticut River population shows plastic responses in transcription of the same enzyme in response to TFM. The similarities in genetic responses between the two treated populations suggest that the TFM‐mediated selection results in a convergent, genetic response.

Two additional genes, *NDUFA9* and *PLCB1,* show genetic signatures of a response to TFM‐mediated selection (Figure 3, Figure 4). A nearly identical variant of *PLCB1*, *PLCD4*, plastically increases expression in Lake Michigan and (albeit not significantly) in Connecticut River in response to TFM (Figure 4e), whereas the expression of *PLCD4* does not respond to TFM in Lake Champlain. The observation that *NDUFA9* was not identified as a genetic outlier in comparisons between Lake Champlain and Connecticut River and that *PLCD4* does not change expression in the presence of TFM in Lake Champlain suggests that modifications to the ETC complex I are more extensive in Lake Michigan, which has been treated with TFM for a longer period of time. Since neutral process alone would be unlikely to affect multiple genes from a metabolic pathway in a coordinated fashion, we suggest that the outlier loci reflect a genetic response to TFM treatment. Moreover, the kNN‐based genome scans suggest that the genetic differentiation between populations at these genes (i.e., *NDUFA9*, *PLCB1*, *ATP5PB*) are likely to be driven by a response to selection (Appendix S1: Figure S11).

Lake Michigan and Lake Champlain have been treated with TFM for 56 and 26 years, respectively. Both show evidence of selection at *ATP5PB*, however, both responded to TFM differently at the gene expression level with 365 and 34 genes differentially expressed in Lake Michigan and Lake Champlain, respectively. These large differences in the number of genes differentially expressed may reflect different evolutionary responses to TFM, one of which is mediated exclusively at the genomic level by shifts in allele frequencies and one that is mediated at both genomic and transcriptomic levels through allele frequency changes and differential gene expression. Alternatively, these differences may reflect the deeper evolutionary histories between these two populations or the different duration and intensities of pesticide usage (Whitehead et al., 2017). These results illustrate that the different evolutionary and treatment histories of Lake Michigan and Lake Champlain can affect overall patterns of gene expression.

While we identified large genetic and transcriptomic differences among populations, we did not detect any individuals able to survive concentrations of TFM applied to streams in practice (Figure 2). The maximum concentration of TFM applied in the field ranges from 5.5 to 9 mg/L, and no larvae were able to survive concentrations equal to or greater than 3 mg/L. Lethal concentration values obtained from our study are similar to estimates from previous studies (see Figure 2 in Dunlop et al., 2017). In 2016, we observed statistically significant, but possibly not biologically meaningful, differences in survivorship among populations over time (*p*‐value = .019) where post hoc pairwise comparisons using the log‐rank test revealed that individuals from Connecticut River population had slightly higher survivorship than individuals from either Lake Michigan or Lake Champlain populations (*p*‐value = .031). However, these slight differences in survivorship only translated to three additional Connecticut River individuals surviving until the end of the experiment (out of 168). In 2017, the slightly lower LC25 and LC50 for larvae collected from Lake Michigan (Figure 2b) suggest that Lake Michigan larvae are more sensitive to TFM than Connecticut River larvae. However, we believe that this result is more likely due to slight differences in body condition (e.g., body size, metabolic rate, and life history stage) between the two populations (Tessier et al., 2018). In our 2017 trials, Connecticut River individuals were slightly larger than individuals collected from Lake Michigan (Appendix S1: Figure S12). Regardless, no larvae from Lake Michigan and Lake Champlain were able to survive at concentrations anywhere close to the levels of TFM that are applied in the field (Figure 2b). This result suggests that outright and widespread resistance has not yet developed in populations treated with TFM.

One possible explanation for this result is that evolutionary constraints and/or reduced selection intensity may be preventing the evolution of full‐fledged resistance in this system. While we have identified potential candidate genes that may illustrate sea lamprey's resistance to TFM, we still lack data associating candidate genes with functional changes or linking genotypic to phenotypic variation. Selection due to environmental factors or other evolutionary forces (e.g., genetic drift) may also contribute to genetic and transcriptomic differences we detected among these populations. Furthermore, we cannot exclude the possibility that some changes in gene expression could be due to maternal effects or heritable epigenetic effects, and in some cases, populations exposed to toxic pollutants may exhibit maladaptive transcriptional responses in which tolerant populations show a higher level of transcription than sensitive populations (Reid et al., 2016).

Nevertheless, the large genetic and transcriptomic differences we report coupled with their role in TFM’s mode of action suggest that we may be documenting the earliest stages of resistance evolving in treated populations (i.e., incipient resistance). Under this model, survival and reproductive advantages conferred by previous generations of larvae exposed to sublethal concentrations of TFM, for example individuals at the peripheries of TFM treatment or individuals buried deeper in the sediment, are inherited by their offspring and have spread throughout the treated populations. It may only be a matter of time before further mutations or a continued response to selection allows individuals to survive higher concentrations of TFM. In fact, recent modeling efforts have revealed that given the estimated strength of selection imposed by lamprey control, full‐fledged resistance to TFM is predicted to occur within the next tens of years (Christie et al., 2019). Because of the high gene flow and lack of population structure found throughout the Great Lakes, fully resistant individuals may already be spreading throughout the population even though it may take many more years for successful detection (Christie et al., 2019). From a pragmatic standpoint, the detection of incipient resistance (IR) means that the development of alternative control measures and the implementation of adaptive management strategies focusing on delaying resistance should be a priority for the continued control of invasive sea lamprey. The detection of resistance in its earliest stages, as suggested here, allows time for such actions to assist with the continued restoration of native and commercially important fishes. We think it would be prudent to continue investigating the resistance of sea lamprey to TFM at multiple levels (i.e., genomic, transcriptomic, and organismal). Regardless of the system, the detection of incipient resistance can greatly assist in efforts to mitigate and delay the costly ecological and economic consequences of resistance.

## 5 COMPETING INTERESTS

The authors declare no competing interests.

## AUTHOR CONTRIBUTIONS

MRC, ASM, and MSS designed the project, all authors provided logistic support, ASM performed molecular analyses, XY, AMH, and ASM performed gene expression and genomic analyses, ASM and MMS performed survival analyses, and XY, ASM, and MRC wrote the manuscript with input from all authors.

### DATA ARCHIVING STATEMENT

Code and scripts are available at https://github.com/ChristieLab/sea_lamprey_TFM. The fully annotated transcriptome assembly and trimmed reads are available via https://www.ncbi.nlm.nih.gov/sra with BioProject accession number PRJNA667554.

## Supporting information

Appendix S1
